# Potential chromosomal introgression barriers revealed by linkage analysis in a hybrid of *Pinus massoniana *and *P. hwangshanensis*

**DOI:** 10.1186/1471-2229-10-37

**Published:** 2010-02-25

**Authors:** Shuxian Li, Ying Chen, Handong Gao, Tongming Yin

**Affiliations:** 1 Jiangsu Key Laboratory for Poplar Germplasm Enhancement and Variety Improvement, the Key Lab of Forest Genetics and Biotechnology, Nanjing Forestry University, Nanjing, China

## Abstract

**Background:**

Exploring the genetic mechanisms underlying speciation is a hot topic in modern genetics and evolutionary studies. Distortion of marker transmission ratio is frequently ascribed to selection against alleles that cause hybrid incompatibility. The natural introgression between *P. massoniana *and *P. hwangshanensis *and their distribution ranges lead to the emergence of the two species as desirable organisms to study the genetic mechanisms for speciation.

**Results:**

Using seeds sampled from trees at different elevations, we consistently detected sharp decreases in seed germination rates of trees in the hybrid zone, which might be due largely to the hybrid incompatibility. A genetic map was established using 192 megagametophytes from a single tree in the hybrid zone of the two species. Segregation distortion analysis revealed that the percentage of significant-segregation-distortion (SSD) markers was extremely high, accounting for more than 25% of the segregating markers. The extension range, the distortion direction, and the distortion intensity of SSD markers also varied dramatically on different linkage groups.

**Conclusions:**

In this study, we display the potential chromosomal introgression barriers between *P. massoniana *and *P. hwangshanensis*. Our study provides a valuable platform for conducting genome-wide association of hybrid incompatible QTLs and/or candidate genes with marker transmission ratio distortion in the hybrid.

## Background

A biological species is defined as a group of natural populations that mate and produce offspring with one another, but do not breed with other populations. Yet biologists have argued over the details of the definition since around 1900[1]. Inter-specific hybridization is a common natural scenario observed both in plants and animals, which roughly occurs in 10% of animal species and 25% of plant species [2]. Inter-specific mating may lead to introgression [3]. Introgression can have various consequences [4]. At one extreme, introgression may cause merging of the hybridization species; at the other extreme, introgression may lead to selection for conspecific mating, and consequently enlarge the reproductive isolation [5]. Early studies suggested that hybrids acted as introgression filters, allowing beneficial genes to filter through and preventing introgression of negative genes [6-8]. Based on these observations, the beneficial genes would have a higher transmission ratio than the negative genes in the offspring of the hybrids. Genetic mapping offers us a powerful tool to display the chromosomal segments that unevenly transmit to the offspring based on marker segregation distortion [9].

*P. hwangshanensis *and *P. massoniana *are desirable organisms to study the genetic mechanism triggering speciation. *P. hwangshanensis *is a native representative conifer that distributes in the subtropical mountainous areas in southeast of China, and it is found at higher elevation than *P. massoniana*. The ranges of the two species are frequently found to be immediately adjacent to each other, and overlapped with a narrow hybrid zone. The two species are different in morphological, cytological and timber anatomical characteristics, and show clear environmental and spatial separation [10-13]. Trees in hybrid zone possess intermediate characteristics. Natural hybridization between the two species has been verified by molecular markers [14]. The major difference in the ecological niches of the two species is elevation. With an increase in elevation, environmental factors, such as oxygen partial pressure, air temperature and moisture regime, soil temperature and water regime, sunray and ultraviolet light intensity, will change [15]. These environmental factors are closely related to plant growth and fitness. They are environmental stresses to cause differentiation in plant phenology and fitness, subsequently, to maintain the species-specific characteristics of the alternate speices. For example, with the change in flowering time, plants will become self-pollinating. Besides divergence in phenology, genetic and cytoplasmic incompatibilities are also important introgression barriers. Genetic incompatibility between species arises in several ways [3]. For instance, pollen and stigma may possess surface proteins that either prevent fusion of the egg and sperm into a zygote, or inhibit pollen tube growth to hamper the fertilization of the plant ovum. Alternatively, once a hybrid zygote is formed, it may have low viability or be sterile [3]. Genetic barriers may also arise through changes in the number of chromosomes in new species [3]. *P. massoniana *and *P. hwangshanensis *are closely related species and they both possess 12 haploid chromosomes. However, there might be some other chromosomal changes between the two species, including chromosomal rearrangement, genome expansion, differential gene expression and gene silencing. These changes may lead to selection for fertility and ecological traits that alter the genome structures of the alternate species, in return acting as introgression barriers [1]. Cytoplasmic incompatibility occurs if the male has an infection that is not present in his mate, resulting in embryonic mortality [2]. All above mechanisms drive conspecies mating. Both conspecies mating and selection of beneficial/negative genes will consequently cause uneven transmission of genetic materials to the progeny from a hybrid parent both in intercross (between hybrid siblings) and backcross (between hybrid and the parental species) situations. Base on marker segregation distortion and linkage analysis in the progeny of a hybrid, we can track the genomic regions that act as introgression barriers. In this paper, we aim to reveal the potential chromosomal introgression barriers between *P. massoniana *and *P. hwangshanensis *by building a genetic map using megagametophytes from a single tree sampled in the hybrid zone and to identify regions of the map displaying extreme segregation distortion.

## Results

### Seed germination test

The germination rates of seeds collected from trees along the transaction lines at different elevations of two different locations were listed in table 1. Early empirical observations revealed that the elevation range of *P. hwangshanensis *was generally above 900 m, and that of *P. Massoniana *was generally below 700 m in the south of the Yangzi River in China, the hybrid zone roughly spanned a vertical range from 700 to 900 m [10-12]. Germination tests showed that seed germination rates of trees in the range of hybrid zone were significantly lower than those of trees sampled outside the hybrid zone. Although we can not tell whether a tree is a hybrid or not merely based on seed germination rate, the consistent low germination rates for many trees in hybrid zone across different locations can not be interpreted by chance alone. We proposed that low seed germination rates for trees in hybrid zone should relate to the hybrid incompatibility. In table 1, there is one tree at 525 m from Wuyi Mountain that also displays a relatively low germination rate. In that sampling area, pines dominate the landscape above 600 m. Below this elevation, pines mix with abundant broad-leave trees, which will affect the pine pollination. We propose the drop in this value might be mainly due to environmental factors. In Table 1, the tree at 795 m in Wuyi Mountain possesses the lowest seed germination rate in the tested samples. However, we did not obtain enough seeds that generated normal seedlings from this tree for it to be used as the mapping parent. Alternatively, we used megagametophytes of seeds from the tree at 784 m as our mapping population.

**Table 1:** The germination rates of seeds from trees sampled at different elevations of two locations.

Location 1(Wuyi)		Location 2(Qimen)	
**Elevation** **(m)**	**Germination rate (%)**	**Elevation** **(m)**	**Germination rate (%)**

525	25.0	396	75.0

646	64.25	451	73.25

758	14.75	700	32.25

784	13.25	753	30.0

795	2.75	824	35.75

1150	75.0	826	34.25

1205	88.25	830	24.0

1235	85.0	870	24.0

		883	33.25

		896	42.75

		1044	75.25

		1081	81.0

Parameters in this table were determined by 400 randomly selected seeds from each tree at the corresponding elevations.

### Marker analysis and segregation test

Twenty-one AFLP primer combinations were selected for this study according to Li *et al*. [16]. Segregating loci were recorded based on presence/absence of the visible alleles. In total, 321 segregating loci were collected from these primer combinations, an average of 15.3 loci per primer pair. Since pines possess gigantic genomes [17], more selective oligonucleotides are needed to reduce the amplified loci in AFLP genotyping. The numbers of selective oligonucleotides used in this study mainly were E+3/M+4 and E+4/M+3(E: *EcoRI*; M; *MseI*; the digital numbers were the number of selective nucleotides). There was considerable variation in the number of segregating loci generated by different primer combinations, ranging from 11 to 33. Based on the Chi-square test, 82 (25.5%) markers were significantly distorted from the expected 1:1 segregation ratio at *α *= 0.05 significance level (corresponding to Chi-square of 3.84). Among them, 37 skewed to more presence and 45 skewed to more absence. The highest Chi-square value of SSD markers is 26.39. Since segregation distortion demonstrates the uneven transmission ratio of alleles on the alternate chromosomes in the mapping parent and is related to hybrid incompatibility [18], once these SSD markers are mapped onto linkage groups, the chromosomal regions acting as potential introgression barriers will be revealed.

### Linkage Map Construction

Linkage analysis with 192 megagametophytes from the hybrid pine was performed with all the obtained 321 segregating AFLP markers. In this paper, all markers that significantly departured from Mendelian segregation ratio were included because these markers were hypothesized to reveal sites of hybrid incompatibility. Using LOD thresholds of 10.0, 7.0, 6.0, 5.0, 4.0, 3.0, 2.0, 321 markers were initially assigned to 19, 18, 16, 16, 16, 9, 3 groups respectively, each run left some ungrouped markers. When LOD≤ 3.0, there were less linkage groups than the haploid chromosome numbers of pine; at LOD = 4.0, 5.0 or 6.0, the same grouping results were derived. Therefore LOD = 6.0 was used to assign markers into linkage groups. Under this criterion, markers were assigned to 14 major linkage groups, 1 triplet, 1 doublet, and 4 unlinked markers. The major linkage groups ranged from 26.9 to 177.9 cM in size (Additional file 1). We did not achieve complete coverage of the pine genome with this map. By gradually decreasing the LOD to 2.0, no strong linkage was detected between the markers that mapped at linkage group ends. Although some loose linkage was observed between markers at the ends of different linkage groups, we did not merge these linkage groups because the loose linkage between markers might have occurred by chance alone. In conclusion, the established map consisted of 14 major linkage groups with a total observed genetic length of 1615.6 cM. Genetic length derived from this study was very close to that of *P. sylvestris *[19] and *P. taeda *[20], both of which were estimated by nearly complete genetic maps. Based on the function (Formula 1 in Methods) given by Lange and Boehnke [21], if we estimated the genetic length of pine ranging from 1500~2000 cM, the estimated coverage of our map would be 95.83~98.62% of the total pine genome, at 10 cM a marker. Therefore, our map achieved nearly complete coverage of the pine genome. The unfilled gaps of this map might be due to presence of recombination hotspots or *EcoRI*/*MseI *restriction deserts in the corresponding genomic regions. Tremendous effort might be needed to fill such gaps with randomly generated markers. The 14 major linkage groups consisted of 312 markers with an average distance of 5.18 cM between the adjacent markers. The established map provides a useful platform for demonstrating the potential chromosomal introgression barriers, and for exploring their expansion ranges on different chromosomes.

### Potential Chromosomal introgression barriers

**Figure 1:**
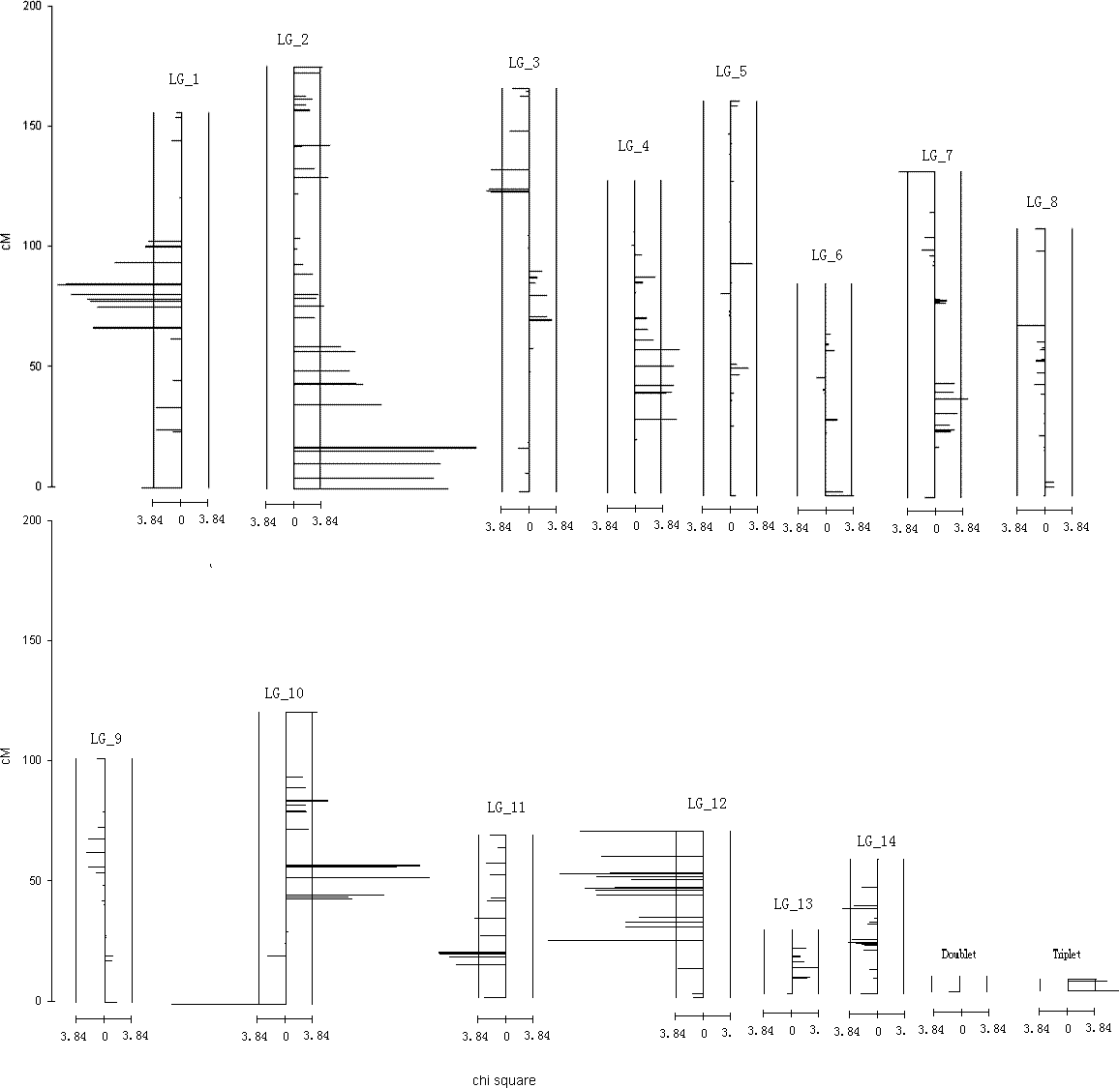
**The distribution, the distortion direction, and the distortion intensity of SSD markers on the established linkage groups**. The vertical rulers at the left indicate the genetic lengths of the linkage groups in cM. The horizontal rulers at the bottom of each linkage group are the chi-square rulers indicating the distortion intensity. In the chart of each linkage group, the left and the right vertical bars corresponding to the Chi-square value of 3.84, which is the statistical criterion to indicate that the segregation distortion does not occur by chance alone; the middle vertical bar corresponding to Chi-square value of 0.00; horizontal bars on each linkage group are used to indicate the position, the distortion direction, and the distortion intensity of the mapped markers; horizontal bars at the left of each middle vertical bar indicate more absence of the visible alleles; horizontal bars at the right of each middle vertical bar indicate more presence of the visible alleles.

Early mapping studies in pine and poplar revealed that the percentage of SSD markers commonly accounted for less than 10% of the segregating markers [19,22,23]. In this study, percentage of SSD markers was found to be extremely high, accounting for more than 25% of the segregating loci, which implied extensive hybrid incompatibilities between the alternate species. The genomic regions, the expansion range, and the distorted direction of these markers were displayed in Figure 1. In this figure, 82 SSD markers were mapped onto 12 linkage groups, including 11 major linkage groups and a triplet. Only one SSD marker remained unlinked. Furthermore, SSD markers were found to be clustered (regions contained three or more SSD markers in a row) on six of the major linkage groups. These SSD marker clusters totally covered 206.7 cM, accounting for 12.8% of the total observed genetic length. Some of these clusters were found to extend to large regions on the corresponding linkage groups, for example, on linkage group 2, it at least covered a genetic length of 59 cM (more than 30% of the genetic length of this linkage group); on linkage group 12, it extended to a genetic length about 46 cM (about 65% of the observed length of this linkage group). Recombination will relax the segregation distortion of a marker caused by its linkage with deleterious genes. Under the observed highest selection intensity, the expansion range of each segregation distortion cluster was estimated by using algorithm 6 in the Methods. It was noteworthy that the observed expansion ranges were much larger than the estimated expansion ranges on most of the linkage groups (Table 2). Since the expansion range was estimated by having the prior that only one locus caused segregation distortion in each SSD cluster, these inconsistencies implied the expansion ranges of most SSD clusters might be triggered by more than one genetic locus, besides, inter-chromosomal epistatic effect might also play a role in the observed inconsistencies.

**Table 2:** The estimated and the observed expansion ranges of the major SSD clusters.

Linkage group	*χ*_*h*_^2^	*o*	*e*	*r*	The estimated expansion range (cM)	The observed expansion range (cM)
1	17.89	65	94	0.082763	16.7	35.9

2	26.39	131	95.5	0.114944	23.4	59.2

3	6.08	78	95	0.018353	3.7	9.2

4	6.48	112	94.5	0.021309	4.3	29.6

10	19.48	126	95.5	0.088775	17.9	14.9

11	9.68	117	95.5	0.041664	8.4	4.9

12	20.67	62	93	0.094856	19.2	46.1

Parameters of *χ*_*h*_^2^, *o*, *e*, and *r *are described in Methods.

## Discussion

Speciation of *P. massoniana *and *P. hwangshanensis *might be the result of parapatric speciation process. Parapatric speciation is one of the evolutionary processes underlying speciation. Then grass species *Anthoxanthum *has been known to undergo parapatric speciation as mine contamination of an area, which creates a selection pressure for tolerance to metals [24]. The main difference between parapatric speciation and sympatric speciation is that, in the former case, two species occupy separate ecological niches and overlap with a narrow hybrid zone. However, during evolutionary time, *P. massoniana *and *P. hwangshanensis *might distribute in separate ranges, thus we can not exclude the hypothesis that their speciation could be due to historical geographic barriers. Although the speciation process of *P. massoniana *and *P. hwangshanensis *is debatable, their ranges and their natural gene introgression make them valuable organisms to exploring the genetic mechanism underlying speciation. Natural recombinants found in hybrid zones will permit genetic mapping of species differences and reproductive barriers in non-model organisms [1]. Pines possess gigantic genomes, which are about 10 fold that of the human genome and about 40 fold that of the poplar genome. Although sequence capacity has increased dramatically with the advent of the next generation sequencing technology, whole genome sequencing for organisms with such huge genomes is not feasible in the near future. In contrast to their huge physical length, genetic lengths of pine genomes were found to be modest which ranged approximately from 1500~2000 cM [19,20]. Thus, it is easy to fast build a genetic map with a good coverage of the pine genome with only a relatively small amount of experimental effort. Linkage analysis, combined with marker segregation distortion analysis, will enable us to display the chromosomal segments that unevenly transmit to the offspring from the mapping parent. Linkage analysis has been applied in studies of many organisms to help our understanding of the genetic mechanisms underlying speciation [9,18,25].

Pine megagametophytes are developed from the megaspore of the maternal tree. In gymnosperms, a diploid precursor cell (megasporocyte or megaspore mother cell) undergoes meiosis to produce four haploid cells, then three of those cells degenerate, results in one functional megaspore per ovule. The megaspore then undergoes megagametogenesis to give rise to the megagametophyte and to produce the female gamete. Thus, the megagametophyte is haploid tissue and has the same DNA as the female gamete that is fertilized by pollen to form embryo in each seed. As a result, marker distortion revealed by megagametophyte genotyping is closely related to selection of alleles on the alternate chromosomes in the maternal parent. Since megagametophytes are haploid, in mapping studies, they possess the similar characteristics as recombination inbreeding lines obtained by the single seed descent method that widely used in the establishment of mapping pedigree for crops.

Seed germination rates reflect the reproductive ability of trees sampled at different elevations. In order to make seed germination rates comparable, seeds from each tree were randomly selected in the test. We did not cull out the empty seeds or seeds with congenital dysplasia embryos. Thus, the low germination rates of trees in hybrid zone could be the result of both pre- and post-zygotic barriers. Pre-zygotic barriers mainly include factors associated with pollination and flowering time, and also include factors affecting process of gamete union to form a zygote. Once a hybrid zygote is formed, it may have low viability or be sterile, and the underlying factors are known as post-zygotic barriers [3]. To resolve the segregation distortion barriers to individual genes will be extremely difficult in pine. First, pines possess gigantic genomes. Early linkage analyses indicated that the expectation numbers of crossovers were only about 15~20 per meiosis in pines [19,20]. One centiMorgan pine genome contains about 500~1000 Mb of nucleotides on average. Second, genes may have the same or opposite directional effect on segregation distortion. Third, besides the underlying genes, segregation distortion can be arisen by epistatic effect, such as linkage disequilibrium (LD) among unlinked markers. Finally, segregation distortion observed in hybrids can also be arisen by chromosomal rearrangements in the parental species. Therefore, the underlying mechanisms vary greatly for each observed SSD clusters. Nevertheless, SSD clusters on a genetic map revealed the genomic regions we should focus on to explore the genetic mechanisms underlying speciation. Although the gigantic genome size of pine hampers resolving the underlying genes, its modest genetic length enables us to detect linkage and map QTL easily. To explore the genetic loci underlying segregation distortion, genome-wide associations between hybrid sterility QTL and marker transmission ratio distortion is a desirable approach [18], and candidate gene approach is a good option to help resolve the underlying genes.

## Conclusions

In this paper, we consistently detected low germination rates of seeds collected from trees in the hybrid zone of *P. massoniana *and *P. hwangshanensis*. We proposed that germination rate reflected the hybrid incompatibility of the alternate species. By using megagametophytes from a single tree in the hybrid zone, we built a nearly complete genetic map for pine genome. Based on the SSD markers on this map, we discovered the potential chromosomal introgression barriers between *P. massoniana *and *P. hwangshanensis*. This study provided the basis for associating SSD markers with their introgression behavior in natural stands, and established a useful platform for conducting genome-wide associations of hybrid sterility QTL and/or candidate genes with marker transmission ratio distortion in the progeny of a hybrid pine.

## Methods

### Cone collection and seed germination

One set of samples used in this study were collected from Huang-gang Chimney in Wuyi Mountain of Fujian province. The elevation range was from 445~1250 m. The other set of samples were collected from Kulongjiang Chimney at Qimen in Anhui province. The elevation range was from 350~1100 m. Linear transects were set up from the foot to the top of the mountains at both locations. To avoid significant geographic changes in horizontal direction, from foot to top, cones were collected from trees within 10 m from the transaction lines. Altogether, cones from 8 trees at 8 elevations were collected in Fujian, and cone from 12 trees at 12 elevations were collected in Anhui. Elevation of each tree was recorded by its GPS reading.

Seed germination was conducted according to "Woody plant seeds inspection standard" in the Chinese Southern Forest Seed Inspection Center [26]. For each tree, 400 seeds were randomly selected to test the germination rate. The germination rate was calculated by ; n was the number of germinating seeds within certain amount days (days were determined based on observation that germinating seeds were less than 1% of the total test seeds in 3 continuous days); and N was the total number of seeds tested for each tree.

### DNA extraction, AFLP genotyping, marker segregation test, and linkage analysis

We selected a tree at 784 m in Wuyi Mountain and harvested the megagametophyte tissues from seeds of this tree that germinated into normal seedlings. DNAs from 192 megagametophytes were extracted as described by Yin *et al *[27]. AFLP primer combinations were selected based on the results in Li *et al *[16]. AFLP genotyping protocol was described by Yin *et al *[19]. AFLP marker nomenclature was designated by using the abbreviation of the restriction enzyme (E: *EcorI*, M: *MseI*), in addition to the number of selective oligonucleotides, following the approximate allele size of the segregating marker. In the name of each primer combination, E primer was before the "/", and M primer was after the "/".

The Chi-square test was performed to check whether a marker segregated in 1:1 ratio. The linkage analysis was conducted by MapMaker version 3.0 [28], and map construction was described as in Yin *et al *[19]. Map charts were drawn with the program of MapChart 2.1 [29]. Genome coverage was estimated by the function given by Lange and Boehnke [21], assuming a random marker distribution,  (1), where *c *was the proportion of the genome within *d *cM of a marker,  was the estimated genome length and *m *was the number of markers.

### Estimate expansion of segregation distortion

If we define the selection intensity (*S*) that causes marker segregation distortion as *S *= |*o *- *e*|, then the Chi-square value of each marker in this study is  (2), where, *o *is the observed number of individuals with a visible allele, *e *is the expect number of individuals with the corresponding visible allele, and *N *is the number of megagametophytes genotyped by the corresponding primer combination. If we assume there is only one locus that causes marker segregation distortion in each cluster, the Chi-square value of a marker that has recombination rate *r *with the driven locus would be  (3). Recombination rate can be derived based on the difference of Chi-square value (*χ*_*d*_^2^) of the two loci, and  (4). Then recombination rate can be transferred into genetic distance by Kosambi's formula [30] as,  (5), where *M *is the genetic distance in Kosambi centiMorgan. Under single driven locus assumption, the two-directional distortion range under the observed highest selection intensity (related to the observed highest Chi-square value, *χ*_*h*_^2^) would be  (6), where *χ*_*d*_^2 ^= *χ*_*h*_^2^-3.84.

## Authors' contributions

LSX, CY and GHD conducted most of the molecular work and data analyses. LSX drafted the manuscript. YTM conceived the work and edited the manuscript critically. All authors have read and approved the final manuscript.

## Supplementary Material

Additional file 1 **Genetic map for a natural hybrid of *P. massoniana *and *P. hwangshanensis***. This genetic map is determined by using megagametophytes of 192 normally germinated seeds from the mapping parent. Marker with name ending with 'r' was in repulsion linkage phase. Click here for file
