# Supplementary figures and images for: Potential chromosomal introgression barriers revealed by linkage analysis in a hybrid of Pinus massoniana and P. hwangshanensis
Source: BMC Plant Biol. 2010 Feb 25;10:37. doi: 10.1186/1471-2229-10-37 (PMC2844070; doi:10.1186/1471-2229-10-37)

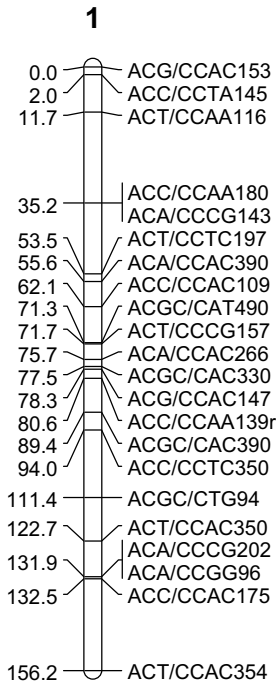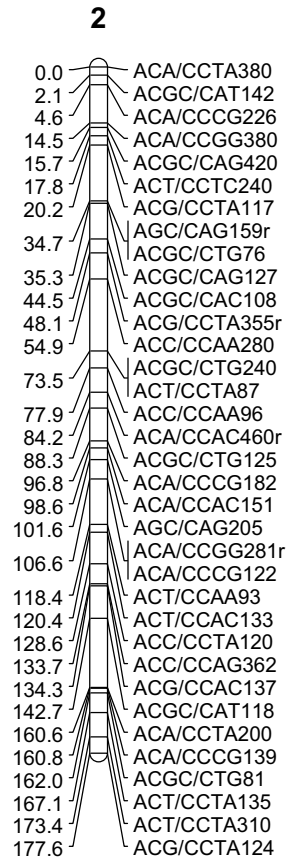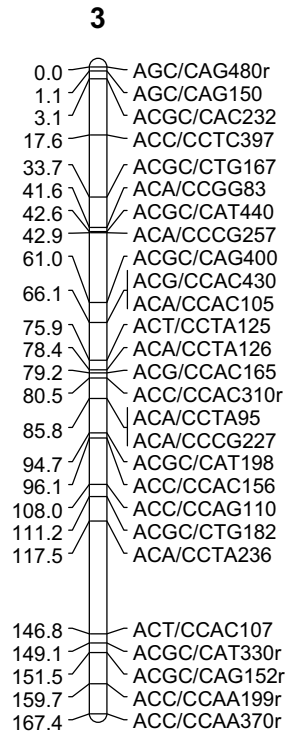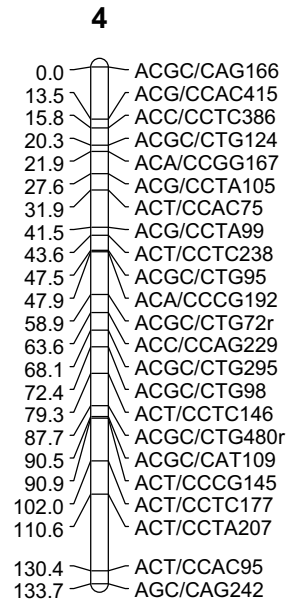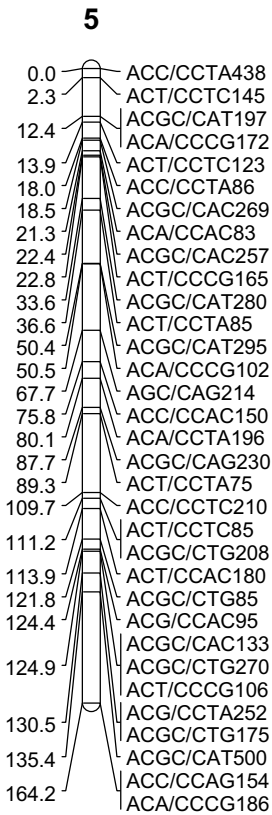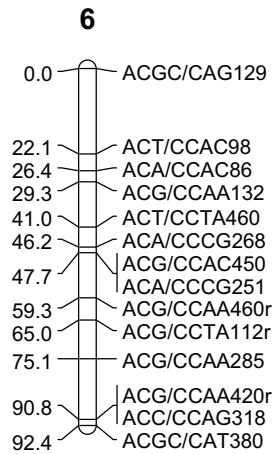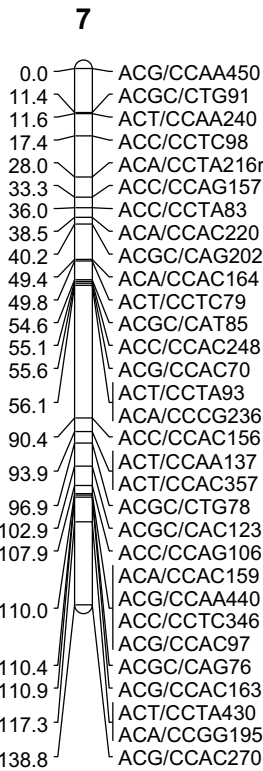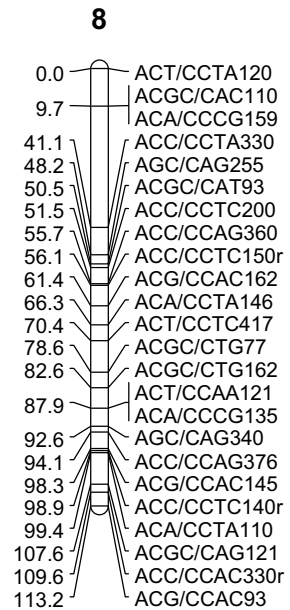

9

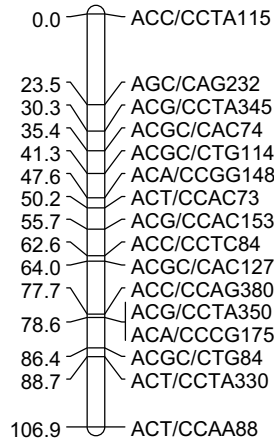

10

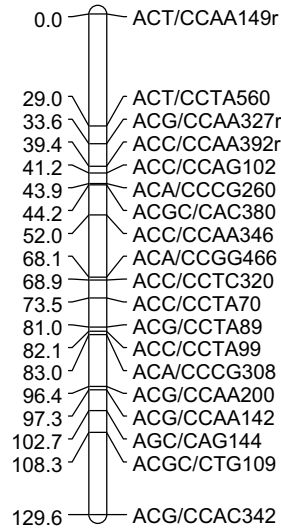

11

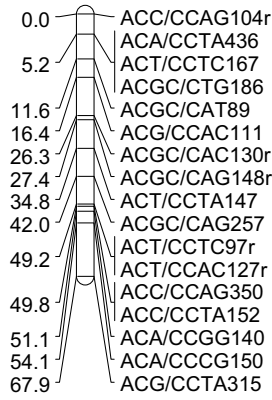

12

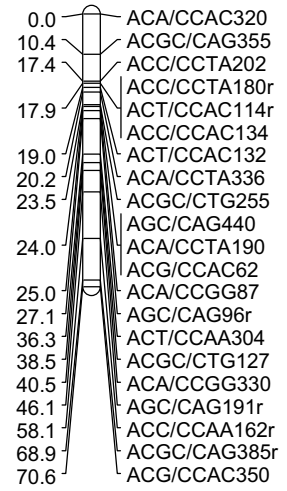

13

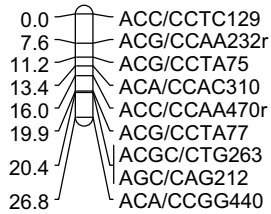

14

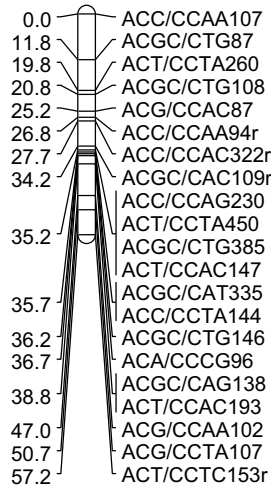

15

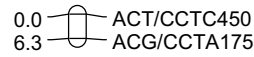

16

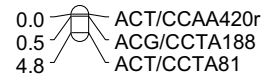

Supplement: Additional file 1 — Genetic map for a natural hybrid of P. massoniana and P. hwangshanensis. This genetic map is determined by using megagametophytes of 192 normally germinated seeds from the mapping parent. Marker with name ending with 'r' was in repulsion linkage phase. [file 1471-2229-10-37-S1.PDF]
